# Acute cardiac functional and mechanical responses to isometric exercise in prehypertensive males

**DOI:** 10.14814/phy2.13236

**Published:** 2017-04-05

**Authors:** Jamie M. O'Driscoll, Katrina A. Taylor, Jonathan D. Wiles, Damian A. Coleman, Rajan Sharma

**Affiliations:** ^1^ School of Human and Life SciencesCanterbury Christ Church UniversityKentUK; ^2^ Department of CardiologySt George's Healthcare NHS TrustTootingLondonUK

**Keywords:** Cardiac function, isometric exercise, ventricular mechanics

## Abstract

Isometric exercise (IE) training has been shown to reduce resting arterial blood pressure (ABP) in hypertensive, prehypertensive, and normotensive populations. However, the acute hemodynamic response of the heart to such exercise remains unclear. We therefore performed a comprehensive assessment of cardiac structure, function, and mechanics at rest and immediately post a single IE session in 26 male (age 44.8 ± 8.4 years) prehypertensive participants. Conventional echocardiography recorded standard and tissue Doppler measures of left ventricular (LV) structure and function. Speckle tracking echocardiography was used to measure LV global longitudinal, circumferential, and radial strain and strain rate. From this data, apical and basal rotation and rotational velocities, LV twist, systolic twist velocity, untwist velocity, and torsion were determined. IE led to a significant post exercise reduction in systolic (132.6 ± 5.6 vs. 109.4 ± 19.6 mmHg, *P *<* *0.001) and diastolic (77.6 ± 9.4 vs. 58.8 ± 17.2 mmHg, *P *<* *0.001) blood pressure, with no significant change in heart rate (62 ± 9.4 vs. 63 ± 7.5b·min^−1^, *P *=* *0.63). There were significant reductions in LV end systolic diameter (3.4 ± 0.2 vs. 3.09 ± 0.3 cm, *P *=* *0.002), LV posterior wall thickness (0.99 ± 0.1 vs. 0.9 ± 0.1 cm, *P *=* *0.013), relative wall thickness (0.4 ± 0.06 vs. 0.36 ± 0.05, *P *=* *0.027) estimated filling pressure (E/E' ratio 6.08 ± 1.87 vs. 5.01 ± 0.82, *P *=* *0.006) and proportion of participants with LV concentric remodeling (30.8% vs. 7.8%, *P *=* *0.035), and significant increases in LV ejection fraction (60.8 ± 3 vs. 68.3 ± 4%, *P *<* *0.001), fractional shortening (31.6 ± 4.5 vs. 39.9 ± 5%, *P *<* *0.001), cardiac output (4.3 ± 0.7 vs. 6.1 ± 1L·min^−1^, *P *<* *0.001), and stroke volume (74.6 ± 11 vs. 96.3 ± 13.5 ml, *P *<* *0.001). In this setting, there were significant increases in global longitudinal strain (−17.8 ± 2.4 vs. −20 ± 1.8%, *P *=* *0.002) and strain rate (−0.88 ± 0.1 vs. −1.03 ± 0.1%, *P *<* *0.001), basal rotation (−5 ± 3.5 vs. −7.22 ± 3.3°, *P *=* *0.047), basal systolic rotational velocity (−51 ± 21.9 vs. −79.3 ± 41.3°·s^−1^, *P *=* *0.01), basal diastolic rotational velocity (48.7 ± 18.9 vs. 62.3 ± 21.4°·s^−1^, *P *=* *0.042), LV twist (10.4 ± 5.8 vs. 13.8 ± 5°, *P *=* *0.049), systolic twist velocity (69.6 ± 27.5 vs. 98.8 ± 35.8°·s^−1^, *P *=* *0.006), and untwist velocity (−64.2 ± 23 vs. −92.8 ± 38°·s^−1^, *P *=* *0.007). These results suggest that IE improves LV function and mechanics acutely. This may in turn be partly responsible for the observed reductions in ABP following IE training programs and may have important implications for clinical populations.

## Introduction

Systemic arterial hypertension remains a significant global public health problem, which is estimated to affect ≈1 billion individuals worldwide (World, 2011) and is associated with considerable morbidity and mortality. A sustained elevation in arterial blood pressure induces specific compensatory cardiac maladaptations that are associated with poor prognosis, including left ventricular hypertrophy, and systolic and diastolic dysfunction (Aljaroudi et al. 2012; Wan et al. 2014), which through a cascade of poorly defined events, progresses to clinically symptomatic heart failure (Drazner 2011).

International guidelines recommend nonpharmacological intervention, including regular physical activity, salt restriction, and weight loss for the primary and secondary prevention of hypertension (Mancia et al. 2013; Eckel et al. 2014). Evidence indicates an inverse, dose‐dependent relationship between levels of physical activity and cardiovascular disease, with reductions in blood pressure being one proposed mechanism (Eckel et al. 2014). The benefits of regular traditional aerobic training are well documented, with improvements in maximal aerobic capacity, physiological cardiac remodeling with coexistent improvements in systolic and diastolic function (Baggish and Wood 2011), and blood pressure reductions (Whelton et al. 2002). However, when compared to traditional aerobic and resistance training (combined and in isolation), isometric exercise (IE) training has shown greater reductions in arterial blood pressure (ABP) (Cornelissen and Smart 2013; Carlson et al. 2014).

A single bout of IE transiently decreases ABP in minutes to hours following exertion (Millar et al. 2009). The potential mechanisms responsible for this post exercise reduction are unclear, but may include reduced vascular resistance and/or decreased cardiac sympathetic nervous system activity (Millar et al. 2014). Indeed, these acute hemodynamic and cardiovascular responses have been shown to be important mechanisms for the observed reductions in ABP following a program of IE training (Millar et al. 2014; Devereux et al. 2015). However, little is known regarding the effects of IE training on cardiac performance both acutely and chronically. The acute cardiac responses to a single IE session may, in part, provide a further mechanistic link to the observed reductions in ABP seen following IE training. Therefore, we performed a comprehensive assessment of cardiac function, including left ventricular (LV) strain, strain rate, rotation and twist at rest and immediately post a single IE training session in a population with prehypertension (pre‐HTN).

## Materials and Methods

### Ethical approval and study population

All procedures for this investigation conformed to the Declaration of Helsinki principles and Canterbury Christ Church Universities Faculty of Social and Applied Sciences Research Ethics Committee approved the study (Ref: 12/SAS/122). Signed, informed written consent was obtained from all participants. We studied 26 physically inactive Caucasian males (age 44.8 ± 8.4 years; height 178.1 **±** 5 cm; body mass 89.9 **±** 1 kg; body surface area 2.1 **±** 1 m^2^) classified as prehypertensive (defined as a blood pressure of 120–139 mmHg systolic and/or 80–90 mmHg diastolic) with no history of cardiac or metabolic disease, nonsmokers currently taking no medication, and with a normal clinical cardiovascular examination and 12‐lead electrocardiogram. We aimed to study a population with pre‐HTN, who were otherwise healthy for three main reasons; first, the homogenous population reduces the impact of other comorbidities on cardiac responses, second, pre‐HTN precedes the development of hypertension, and third, cardiac mechanical responses in this group may provide important mechanistic information for blood pressure reduction, which may prove important for future IE training interventions in hypertensive populations.

## Experimental Procedures

Participants attended the laboratory on three separate occasions each separated by 48 h and were required to fast for 8 h and abstain from caffeine and alcohol for 24 h before testing. All participants were required to maintain their normal circadian and dietary patterns and attend the laboratory at the same time of day. The first session comprised of initial resting blood pressure assessment to confirm pre‐HTN. Each participant was seated for 15 min with the cuff at heart level. After this, three resting automated blood pressure measurements were performed (Dinamap Pro 200 Critikon, GE Medical Systems, Freiburg, Germany) (Reinders et al. 2006) at 5‐min intervals and the average was recorded (Pickering et al. 2005; Mancia et al. 2013).

**Figure 1:**
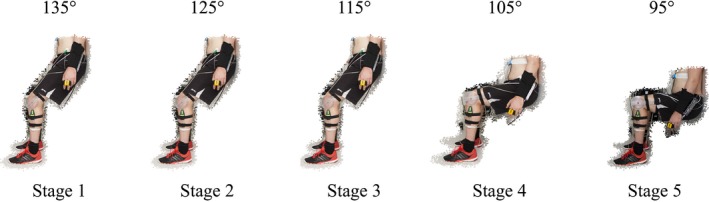
Knee joint angles used for the five consecutive 2‐min stages of the incremental isometric exercise test (left to right: 135°, 125°, 115°, 105°, and 95°).

Prior research has demonstrated that when constant electromyography (EMG) was used to determine exercise intensity, a steady state heart rate was achieved at 10, 15, 20, 25, and 30% EMG (Wiles et al. 2008). This physiological response established the potential for IE training prescription via heart rate. Subsequent to this, research from our laboratory has demonstrated that isometric wall squat intensity could be adjusted by manipulating knee joint angle, which resulted in reliable heart rate responses (Goldring et al. 2014). This method of isometric exercise prescription elicited similar cardiovascular responses to other IE modes and has recently been shown to significantly reduce resting blood pressure (Wiles et al. 2017). As such, IE intensity was determined based on participant heart rate and blood pressure responses to an incremental isometric exercise test (Wiles et al. 2008) using the wall squat as previously described (Goldring et al. 2014). Participants were required to rest their back against a fixed wall with their feet parallel, shoulder width apart, and their hands by their side. Participants were instructed to lower their back down a solid wall, and make small adjustments to their feet position until the required knee joint angle was reached while maintaining a vertical lower limb and an erect trunk. Knee joint angle was measured using a clinical goniometer (MIE Medical Research, Leeds, UK), secured to the participants lower and upper leg using elasticated Velcro strapping. The test consisted of five consecutive 2‐min stages, beginning at a knee joint angle of 135° and guided to reduce the angle by 10° every 2 min (125°, 115°, 105°, and 95°) (Fig. 1). Each participant's feet position was measured from the back of the left heel to the wall and their back position was measured as the distance from the ground to the lower back, which was defined as the lowest point of contact that the participants back had with the wall. Participants were not permitted to stand or rest between angles, and maintained the wall squat until volitional exhaustion or completion of the 10‐min test. Verbal encouragement was given throughout, with particular instructions to maintain normal breathing to avoid the Valsalva maneuver. Rating of perceived exertion (Borg CR10 scale) was recorded at the end of each stage and/or test termination, to obtain a subjective indicator of effort. Heart rate and blood pressure were monitored continuously during the test using a plethysmographic device (Task Force^®^ Monitor, CNSystems, Graz, Austria) to ensure participants remained within safe exercising limits defined by American College of Sports Medicine.

The mean heart rate for the last 30 sec of each completed incremental stage was recorded. Prior research has demonstrated that knee joint angle produced an inverse curvilinear relationship with heart rate (Goldring et al. 2014). As such, knee joint angle was plotted against mean heart rate for the last 30 sec of each stage. The inverse curvilinear relationship produced was used to calculate each participant's knee joint training angle that would elicit a target training heart rate of 95% peak heart rate as used in prior research (Devereux et al. 2010; Wiles et al. 2010).

On the second visit, each participant performed a familiarization IE training session at their prescribed training angle (mean 106 ± 7°), which consisted of four 2‐min isometric wall squats interspersed with 2‐min recovery. On the third visit, each participant repeated the single IE training session at their prescribed training angle and physiological data were recorded pre and immediately post the IE session.

### Conventional echocardiographic image acquisition

Transthoracic echocardiography was performed using a commercially available, portable ultrasound system (Vivid‐q, GE Healthcare, Milwaukee, Wisconsin) with a 1.5–3.6 MHz phased array transducer (M4S‐RS Matrix cardiac ultrasound probe), pre and immediately post the single IE training session. The same sonographer acquired all images, with the participant examined in the left lateral decubitus position. Cardiac structural and functional measurements were recorded as recommended by current guidelines (Lang et al. 2015). Three consecutive cardiac cycles were recorded and stored for offline analysis using commercial software on a proprietary workstation (EchoPAC; V.113.0.x, GE Healthcare), with the results averaged. Images were acquired in parasternal long axis and short axis (level of mitral valve and apex), and apical 2‐, 3‐, 4‐chamber views at baseline (following 15‐min of supine rest) and immediately post exercise. Interventricular septal and posterior wall thickness, fractional shortening, and LV internal dimensions were recorded and relative wall thickness was calculated as (2 × LV posterior wall thickness)/LV internal diameter. LV mass was calculated according to (Devereux et al. 1986) and indexed to body surface area. LV ejection fraction was determined by the modified biplane Simpson's rule. The LV length was measured in the apical 4‐chamber view from the mitral valve plane to the most distal endocardium at the LV apex. Pulsed wave Doppler recordings were obtained to assess transmitral early (E) and late (A) diastolic filling velocities from the apical 4‐chamber view, with the sample volume placed at the tips of the mitral valve. Isovolumic relaxation time was measured from the start of aortic valve closure to mitral valve opening. Tissue Doppler imaging was acquired at the lateral and septal mitral annulus to assess peak longitudinal (S'), peak early diastolic (E'), and late diastolic (A') velocities, with values averaged. LV filling pressure was estimated from the mitral E/E' ratios (Ommen et al. 2000). Stroke volume was calculated by the product of LV outflow tract area and velocity time integral from a pulsed wave Doppler signal placed in the LV outflow tract in an apical 5‐chamber view. Cardiac output was calculated as the product of stroke volume and heart rate.

### Cardiac mechanics: strain, rotation, and twist

**Figure 2:**
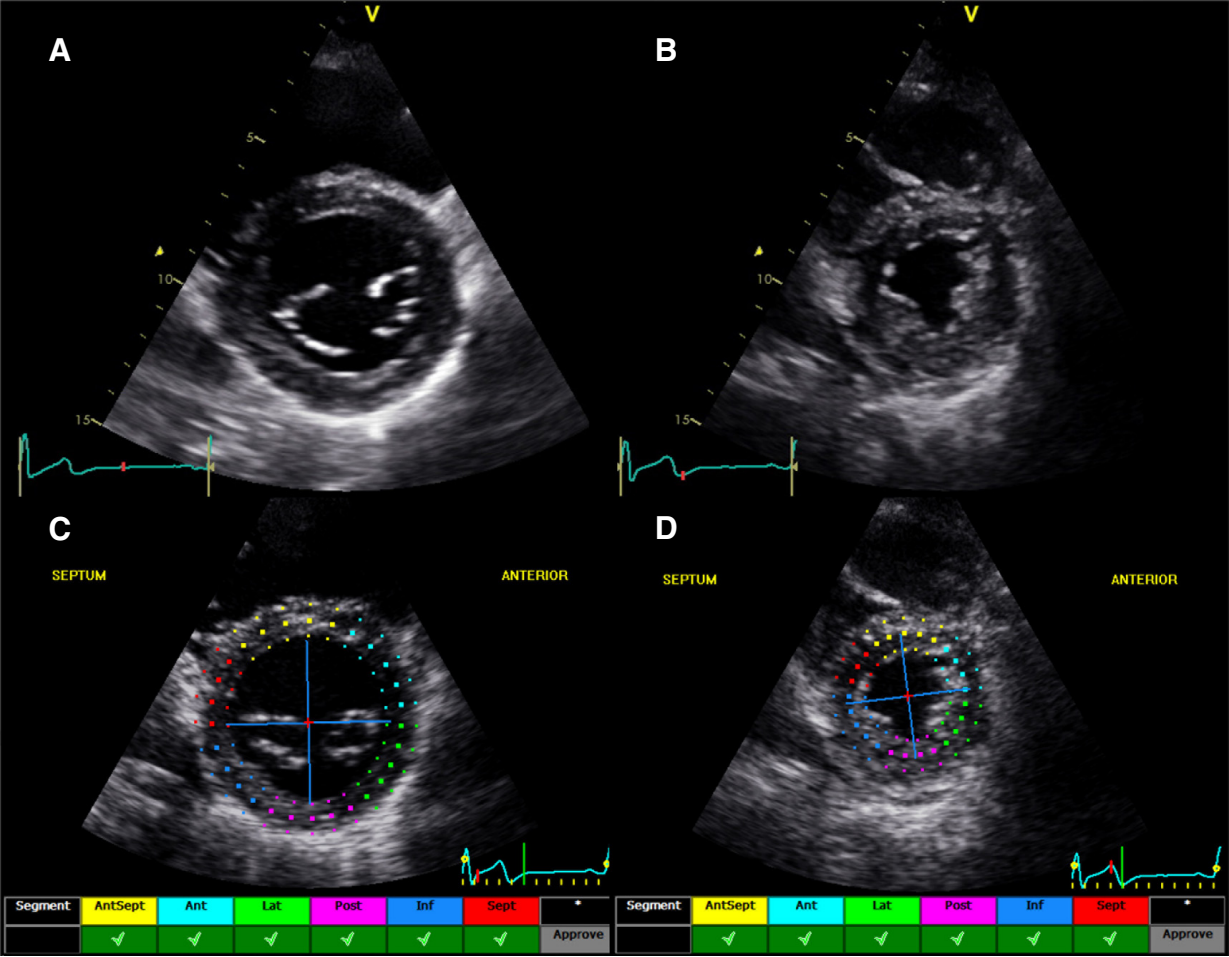
Representative short axis images and speckle tracking imaging. Strict imaging criteria was utilized in order to standardize all parasternal short axis images. (A) An adequate basal image was defined by the presence of full thickness myocardium surrounding the mitral valve at end systole. (B) The left ventricular (LV) apex was obtained by moving the transducer one to two intercostal spaces caudally from the basal position to align with the apical short axis with no visible papillary muscles that closely approximated an end diastolic ratio of LV cavity diameter to total LV diameter of 0.5, as described previously (Weiner et al. 2010). The endocardium was traced manually on the two‐dimensional image and the speckle tracking software automatically tracked myocardial motion and only acceptable tracking was accepted as shown in (C) basal and (D) apical short axis images.

Speckle tracking imaging was used to obtain global LV longitudinal strain and the time‐derivative strain rate from the apical 2‐, 3‐, and 4‐chamber views. LV radial and circumferential strain and strain rate, and LV rotation and rotational velocity were obtained from parasternal short axis views obtained from the LV base at the level of the mitral valve (mitral valve leaflets on view) and the LV apex (circular LV cavity with no papillary muscle visible), as described previously (Leitman et al. 2004; Notomi et al. 2005; van Dalen et al. 2008; Weiner et al. 2010) (Fig. 2). For speckle tracking analysis, the highest quality digital images were selected and the endocardium was traced. A full thickness myocardial region of interest was selected. The observer readjusted the endocardial trace line and/or region of interest width to ensure an acceptable tracking score.

Since basal and apical rotation are not acquired from the same cardiac cycle and to enable comparison between and within subjects, raw frame‐by‐frame rotation and rotation rate data were normalized to the percentage duration of systole and diastole using cubic spline interpolation (GraphPad Prism 6 Software, California) (Borg et al. 2008; Burns et al. 2009; Stembridge et al. 2014). Subtraction of the basal data from the apical data at each time point was undertaken to calculate LV twist and untwist (Borg et al. 2008; Burns et al. 2009; Stembridge et al. 2014). LV torsion was defined as LV twist per unit length and was calculated by dividing the total twist by LV diastolic length. Images were optimized for sector width and scan depth in order to obtain high frame rates (>60 Hz) and kept constant for repeat examinations. Intra‐ and interobserver variability was performed on 12 randomly selected participants and correlation coefficients using linear regression ranged from *r* ^2^ 0.92 to *r* ^2^ 0.95.

### Statistics

Measurements are presented as mean ± standard deviation. All data were analyzed using the statistical package for social sciences (SPSS 22 release version for Windows; SPSS Inc., Chicago IL). A paired sample t‐test was used to compare baseline and post‐IE measurements after confirmation of normal distribution. A chi‐squared test was used to compare categorical data. A *P* < 0.05 was regarded as statistically significant.

## Results

### General

All 26 participants recruited completed the IE training session, which comprised of four 2‐min isometric wall squats at each participant's prescribed knee joint angle, interspersed with 2‐min recovery. Echocardiographic images suitable for complete analysis were obtained on all subjects at rest and immediately post exercise.

### Hemodynamics

There was a significant increase in cardiac output (4.3 ± 0.7 vs. 6.1 ± 1 L·min^−1^, *P *<* *0.001), predominantly mediated via a significant increase in stroke volume (74.6 ± 11 vs. 96.3 ± 13.5 mL, *P *<* *0.001) post exercise, since there was no significant change in heart rate (62 ± 9.4 vs. 63 ± 7.5 b·min^−1^, *P *=* *0.63). In addition, IE was associated with a significant reduction in systolic (132.6 ± 5.6 vs. 109.4 ± 19.6 mmHg, *P *<* *0.001), diastolic (77.6 ± 9.4 vs. 58.8 ± 17.2 mmHg, *P *<* *0.001), and mean ABP (94.7 ± 10.1 vs. 78.8 ± 18 mmHg, *P *<* *0.001) in recovery.

### Cardiac function and structure: conventional and tissue Doppler parameters

Baseline and post IE echocardiographic structural, functional, and tissue Doppler parameters are detailed in Table 1. There was a significant decrease in LV end systolic diameter (3.4 ± 0.2 vs. 3.09 ± 0.3 cm, *P *=* *0.002), LV posterior wall thickness (0.99 ± 0.1 vs. 0.9 ± 0.1 cm, *P *=* *0.013) and relative wall thickness (0.4 ± 0.06 vs. 0.36 ± 0.05, *P *=* *0.027) following the IE training session, with no change in LV end diastolic diameter, interventricular septal thickness or LV length. There was a significant increase in the proportion of participants with normal LV geometry following IE (69.2% vs. 92.2%, *P *=* *0.035).

**Table 1:** Left ventricular function from standard and tissue Doppler echocardiography

Structural parameters	Pre‐IET	Post‐IET	*P* value
LV internal diameter diastole (cm)	4.98 ± 0.4	5.09 ± 0.47	0.42
LV internal diameter systole (cm)	3.4 ± 0.2	3.09 ± 0.3	0.002
IVSd (cm)	0.98 ± 0.1	0.93 ± 0.1	0.16
LVPWd (cm)	0.99 ± 0.1	0.9 ± 0.1	0.013
Relative wall thickness	0.4 ± 0.06	0.36 ± 0.05	0.018
LV mass (g)	177.8 ± 31.7	164.6 ± 26.8	0.16
LV mass index (g·m^2^)	86.3 ± 15	80 ± 13.8	0.18
LV geometry			
Normal	18	24	0.035
Concentric remodeling	8	2	
LV length (cm)	8.9 ± 0.6	8.8 ± 0.7	0.7
Global LV diastolic function			
Peak E velocity (cm·s^−1^)	0.7 ± 0.1	0.74 ± 0.2	0.32
Peak A velocity (cm·s^−1^)	0.5 ± 0.2	0.51 ± 0.2	0.82
Peak E/A ratio	1.48 ± 0.3	1.53 ± 0.4	0.69
Isovolumic relaxation time (ms)	77.2 ± 15	82.1 ± 23	0.67
Global LV systolic function			
Left ventricular ejection fraction (%)	60.8 ± 3	68.3 ± 4	<0.001
Fractional shortening (%)	31.6 ± 4.5	39.9 ± 5	<0.001
Heart rate (b·min^−1^)	62 ± 9.4	63 ± 7.5	0.63
Stroke volume (mL)	74.6 ± 11	96.3 ± 13.5	<0.001
Cardiac output (L·min^−1^)	4.3 ± 0.7	6.1 ± 1	<0.001
LV tissue Doppler			
Average peak E' (m·s^−1^)	0.12 ± 0.02	0.15 ± 0.03	<0.001
Average peak A' (m·s^−1^)	0.1 ± 0.02	0.11 ± 0.02	0.05
Average peak S' (m·s^−1^)	0.09 ± 0.01	0.19 ± 0.04	<0.001
LV filling pressures			
Average E/E' ratio	6.08 ± 1.87	5.01 ± 0.82	0.006
Arterial pressures			
Systolic (mmHg)	132.6 ± 5.6	109.4 ± 19.6	<0.001
Diastolic (mmHg)	77.6 ± 9.4	58.8 ± 17.2	<0.001
Mean (mmHg)	94.7 ± 10.1	78.8 ± 18	<0.001

LV, Left ventricular; IVSd, Interventricular septal thickness diastole; LVPWd, Left ventricular posterior wall thickness diastole.

There was a significant increase in LV ejection fraction (60.8 ± 3 vs. 68.3 ± 4%, *P *<* *0.001) and fractional shortening (31.6 ± 4.5 vs. 39.9 ± 5%, *P *<* *0.001) following IE. There were no significant changes in global diastolic function; however, there were significant increases in LV tissue Doppler S' (0.09 ± 0.01 vs. 0.19 ± 0.04, *P *<* *0.001) and E' (0.12 ± 0.02 vs. 0.15 ± 0.03, *P *<* *0.001), with the latter resulting in a significant decrease in estimated filling pressure post IE (E/E' ratio 6.08 ± 1.87 vs. 5.01 ± 0.82, *P *=* *0.006).

### LV strain, rotation, torsion, and untwisting

**Figure 3:**
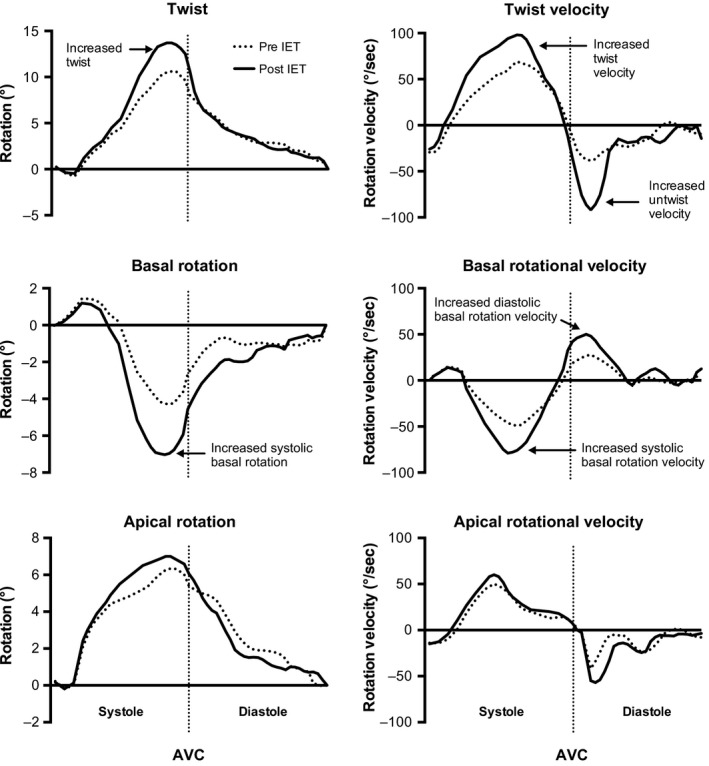
Sequential representation of left ventricular twist, basal, and apical rotation pre and post isometric exercise training. Annotations indicate key findings and for clarity, statistical differences have not been displayed; refer to Table 2. Note: AVC, aortic valve closure.

Myocardial mechanics pre and post IE are displayed in Table 2. Global longitudinal strain (−17.8 ± 2.4 vs. −20 ± 1.8%, *P *=* *0.002) and strain rate (−0.88 ± 0.1 vs. −1.03 ± 0.1%·s^−1^, *P *<* *0.001) significantly increased post IE with no difference in global longitudinal diastolic strain rate (1.26 ± 0.3 vs. 1.37 ± 0.3%·s^−1^, *P *=* *0.259). There was a significant increase in both basal and apical circumferential strain (−28.9 ± 5.4 vs. −34.8 ± 6.3%, *P *=* *0.003 and −25.3 ± 4.1 vs. −32.9 ± 7.6%, *P *<* *0.001, respectively) and strain rate (−2.3 ± 0.5 vs. −2.8 ± 0.6%·s^−1^, *P *=* *0.009 and −2.04 ± 0.5 vs. −2.57 ± 0.7%·s^−1^, *P *=* *0.012) and significant increase in apical radial strain (35.4 ± 16.4 vs. 55 ± 17.8%, *P *=* *0.001). There was a significant increase in basal rotation (−5 ± 3.5 vs. −7.22 ± 3.3°, *P *=* *0.047), basal systolic rotational velocity (−51 ± 21.9 vs. −79.3 ± 41.3°·s^−1^, *P *=* *0.01), and basal diastolic rotational velocity (48.7 ± 18.9 vs. 62.3 ± 21.4°·s^−1^, *P *=* *0.042); however, there was no significant change in apical rotation, apical systolic rotational velocity, and apical diastolic rotational velocity. The increase in basal mechanics translated into a significant increase in LV twist (10.4 ± 5.8 vs. 13.8 ± 5 °, *P *=* *0.049), systolic twist velocity (69.6 ± 27.5 vs. 98.8 ± 35.8 °·s^−1^, *P *=* *0.006), untwist velocity (−64.2 ± 23 vs. −92.8 ± 38 °·s^−1^, *P *=* *0.007), and LV length‐corrected torsion (1.46 ± 0.86 vs. 2.07 ± 0.88 °·cm^−1^, *P *=* *0.032). Figure 3 displays the composite twist, basal and apical rotation and rotational velocity curves with annotations indicating key findings.

**Table 2:** Myocardial mechanics pre‐ and postisometric exercise training

	Pre‐IET	Post‐IET	*P* value
LV longitudinal parameters			
Peak global LV longitudinal strain (%)	−17.8 ± 2.4	−20 ± 1.8	0.002
Peak global LV longitudinal strain rate (%·s^−1^)	−0.88 ± 0.1	−1.03 ± 0.1	<0.001
Peak global LV longitudinal strain rate diastole (%·s^−1^)	1.26 ± 0.3	1.37 ± 0.3	0.259
LV basal parameters			
Basal rotation (°)	−5 ± 3.5	−7.22 ± 3.3	0.047
Basal systolic rotational velocity (°·s^−1^)	−51 ± 21.9	−79.3 ± 41.3	0.01
Basal diastolic rotational velocity (°·s^−1^)	48.7 ± 18.9	62.3 ± 21.4	0.042
Basal radial strain (%)	48.6 ± 22.9	55.5 ± 19.4	0.305
Basal radial strain rate (%·s^−1^)	3.3 ± 1.2	3.9 ± 1.8	0.205
Basal circumferential strain (%)	−28.9 ± 5.4	−34.8 ± 6.3	0.003
Basal circumferential strain rate (%·s^−1^)	−2.3 ± 0.5	−2.8 ± 0.6	0.009
LV apical parameters			
Apical rotation (°)	6.58 ± 4.5	7.8 ± 4.4	0.389
Apical systolic rotational velocity (°·s^−1^)	52.1 ± 22.1	60.5 ± 26	0.278
Apical diastolic rotational velocity (°·s^−1^)	−42.2 ± 18.3	−57.9 ± 31.7	0.062
Apical radial strain (%)	35.4 ± 16.4	55 ± 17.8	0.001
Apical radial strain rate (%·s^−1^)	3 ± 1.6	3.6 ± 1.5	0.17
Apical circumferential strain (%)	−25.3 ± 4.1	−32.9 ± 7.6	<0.001
Apical circumferential strain rate (%·s^−1^)	−2.04 ± 0.5	−2.57 ± 0.7	0.012
LV twist parameters			
Twist (°)	10.4 ± 5.8	13.8 ± 5	0.049
Systolic twist velocity (°·s^−1^)	69.6 ± 27.5	98.8 ± 35.8	0.006
Untwist velocity (°·s^−1^)	−64.2 ± 23	−92.8 ± 38	0.007
Torsion (°·cm^−1^)	1.46 ± 0.86	2.07 ± 0.88	0.032

LV, Left ventricular.

## Discussion

To the best of our knowledge, this study is the first to investigate the acute effects of isometric wall squat exercise on cardiac structure, function, and LV mechanics in men with pre‐HTN. A single IE training session was associated with a significantly reduced LV end systolic internal diameter, LV posterior wall thickness, relative wall thickness, and proportion of patients characterized with concentric LV remodeling. These favorable LV remodeling responses to acute IE are similar to those reported in patients with hypertension following a program of aerobic exercise training or prescribed diuretics (Rinder et al. 2004). A single IE training session was associated with improved global longitudinal, circumferential strain and strain rate and apical radial strain as well as increased LV twist and untwist. These favorable responses in cardiac mechanics have been demonstrated in healthy volunteers during aerobic exercise (Notomi et al. 2006). Our results suggest that IE acutely improves LV remodeling, LV systolic and diastolic function, and LV mechanics. These positive adaptive changes may in turn contribute to the observed reductions in ABP following IE training programs (Inder et al. 2016) and have important implications for clinical populations.

The mechanisms responsible for this acute response may in part be due to the significant increase in cardiac systolic function and significant decrease in estimated LV filling pressure and LV after‐load. Indeed, Rinder et al. (2004) demonstrated a significant correlation between reductions in systolic blood pressure and reduced relative wall thickness and reported an increase in stroke volume, cardiac output, and LV ejection fraction in their exercise training group. Other lifestyle interventions, such as low sodium diets have demonstrated significantly improved LV diastolic function with significantly reduced ABP in hypertensive heart failure patients (Hummel et al. 2012, 2013). Although not statistically significant, reductions in LV mass index, relative wall thickness and increased LV ejection fraction, stroke volume, and cardiac output were also reported (Hummel et al. 2013).

LV peak strains and strain rate have been proposed as indicators of regional myocardial function (Greenberg et al. 2002). Our study demonstrated that global longitudinal and radial systolic function improved post IE. This may in part be explained by a maintained pre‐load, as suggested by LV end diastolic dimensions, and increased contractility (decreased LV end systolic dimension), mediating an increase in fractional shortening, LV ejection fraction, and stroke volume. The underlying mechanism may include an increase in excitation–contraction coupling via improved cardiac calcium signaling as a result of sympathetic nervous system activity and/or nitric oxide bioavailability. Nitric oxide has been reported to exert significant effects on cardiac function, in particular LV relaxation and may modulate fundamental events of myocardial excitation–contraction coupling (Paulus and Shah 1999). In addition, a recent animal study demonstrated that dietary nitrate, which is known to reduce blood pressure, improves cardiomyocyte calcium signaling and LV contractile function (Pironti et al. 2016).

An acute IE training session induced a significant increase in LV twist and untwist, primarily mediated by significant increases in basal rotation, basal systolic rotational velocity, and basal diastolic rotational velocity. Similar responses have been described following acute submaximal and maximal aerobic exercise (Drury et al. 2012). Among patients with treated hypertension, reduced and delayed untwisting is reported with worsening LV remodeling, which may contribute to LV relaxation abnormalities (Takeuchi et al. 2007). Enhanced LV twist or torsional deformation augments potential energy during the ejection phase and the recoil of this systolic deformation and release of elastic energy (bidirectional spring) may contribute to pressure decay, enhancing LV suction and associated diastolic filling (Dong et al. 2001; Kass et al. 2004). Studies in human volunteers reported that invasive measures of LV pressure and indexes of LV untwist are related to parameters of early diastolic filling (Burns et al. 2009). These authors reported that reductions in the rate and magnitude of untwisting were associated with worsening early diastolic suction and supported the concept that untwisting is important in generating LV suction and improving early diastolic filling (Burns et al. 2009). In our study, there were no significant differences in conventional measures of diastolic function.

The LV mechanical responses may in part be explained by mechanisms that also result in reduced ABP post IE training interventions. Prior research demonstrated significant improvements in cardiac autonomic regulation (reduced sympathetic activity) and post exercise reductions in ABP, following a single bout of bilateral isometric hand‐grip exercise (Millar et al. 2009). Post exercise hyperemia and associated sheer stress, mediating increased nitric oxide bioavailability are other potential mechanisms (Tinken et al. 2010). Together, these physiological responses reduce peripheral vascular resistance, which reduces cardiac after‐load and improves LV hemodynamics.

Our results contrast the findings of previous research, which utilized the isometric hand‐grip test to induce an increase in LV after‐load and assess LV twist mechanics (Weiner et al. 2012). In this study, isometric hand‐grip exercise produced a significant transient increase in ABP and LV end systolic volume. The increased after‐load induced significant reductions in LV systolic and diastolic function and significant reductions in apical rotation, basal rotation and LV twist and untwist mechanics. However, the authors recorded echocardiographic data during the isometric contraction, as opposed to the recovery period, which was performed in our study. Similar results were reported with a single isometric hand‐grip exercise session followed by a period of post exercise circulatory occlusion (Balmain et al. 2016). Studies by both Weiner et al. (2012) and Balmain et al. (2016) in healthy populations confirm data from clinical populations, where a decrease in cardiac mechanics is associated with an increase in LV after‐load (Takeuchi et al. 2007), which is an important finding when considering the continuum of hypertensive heart disease from raised after‐load to adverse LV remodeling to cardiac failure. It is conceivable that had Weiner et al. (2012) recorded data in the recovery period, they may have produced similar results to this study, since ABP reduced below baseline in the recovery period.

### Study limitations

Our study is limited by a small sample size and comprised only male, Caucasian participants. We acknowledge that maximal IE tension was not recorded due to the fact that the wall squat does not use external resistance as a means of determining exercise intensity. However, the wall squat method used in this study has been shown to produce similar heart rate responses to other lower limb IE protocols and also produce significant reductions in resting blood pressure. Therefore, it is feasible to suggest that participants produced similar tension to that documented in previous IE research where tension equated to approximately 24% maximal voluntary contraction when using 95% heart rate peak (Devereux et al. 2010). Speckle tracking echocardiography has inherent limitations and the exact location of the basal and apical planes may be different from patient to patient. The acute responses highlighted may simply be a consequence of improved LV contractility and reduced after‐load. Nevertheless, the findings are still of significant interest since IE training interventions have been shown to reduce after‐load, which may elicit long‐term improvements in myocardial performance. However, whether these acute responses translate into sustained cardiac adaptations is as yet unknown.

### Clinical perspective

Arterial hypertension can induce a progressive deterioration in cardiac performance and is the leading modifiable risk factor for premature mortality globally (World, 2012). Pre‐HTN is highly prevalent and is associated with a higher incidence of cardiovascular disease compared to optimal blood pressure (Vasan et al. 2001). IE training interventions result in greater reductions in ABP compared with traditional exercise modalities (Cornelissen and Smart 2013) and is a short duration exercise intervention that can be performed in the home. This study demonstrates that a single IE training session results in acute improvements in LV remodeling, LV function and mechanics. The long‐term adaptation of the LV to IE training interventions remains unknown. However, these acute favorable responses provide an insight into the observed reductions in ABP after a period of IE training. These results may in turn be partly responsible for the observed reductions in ABP following IE training programs. Although further work is needed, this study supports the potential role for IE training as a valid treatment for blood pressure lowering.

## Conclusion

A single IE training session was associated with significant changes in cardiac remodeling, function, and LV mechanics in a population with pre‐HTN. The acute cardiac responses seen may be clinically important and help further our understanding of the mechanisms inducing blood pressure reductions and improving cardiovascular health following IE training. Future short‐ and long‐term IE training interventions are needed in order to understand the implications of these acute cardiac responses.

## Conflicts of Interest

No conflicts of interest, financial or otherwise, are declared by the author(s).
